# Evaluating the utility of the Allplex STI Essential Assay to determine the occurrence of urogenital sexually transmitted infections among symptomatic and asymptomatic patients in Cape Town, South Africa

**DOI:** 10.1371/journal.pone.0292534

**Published:** 2023-11-29

**Authors:** Clinton Moodley, Hafsah Tootla, Imaan Amien, Mark E. Engel

**Affiliations:** 1 Division of Medical Microbiology, Anzio Road Observatory, University of Cape Town, Cape Town, South Africa; 2 National Health Laboratory Service, Observatory, Cape Town, South Africa; 3 Department of Medicine, Cape Heart Institute, University of Cape Town, Cape Town, South Africa; GGD Amsterdam, NETHERLANDS

## Abstract

**Background:**

Sexually transmitted infections are among the most commonly occurring infections globally, with countries in sub-Saharan Africa exhibiting disproportionately higher prevalence rates. Numerous reports indicate the need for accurate detection, epidemiological characterisation, and appropriate management of these infections. This prospective observational laboratory study sought to determine the occurrence of STI, using a validated molecular assay as a diagnostic and surveillance tool in our setting.

**Methods:**

Urogenital swabs from symptomatic and asymptomatic patients, submitted to the National Health Laboratory Service, at Groote Schuur Hospital, from 04 August 2021–03 February 2022, for routine microbiological investigations, were subjected to the Allplex™ STI Essential Assay (Seegene Inc, South Korea) to determine the distribution of STI pathogens in our setting. This multiplex assay includes *C*. *trachomatis*, *Mycoplasma genitalium*, *Mycoplasma hominis*, *N*. *gonorrhoeae*, *Trichomonas vaginalis*, *Ureaplasma parvum*, and *Ureaplasma urealyticum*. Correlations between detected organisms and participant age and clinical indications for testing were determined using Stata® software.

**Results:**

A total of 148 urogenital swabs (91.2% from women) were included in the analysis, of which 56/148 (37.84%) were from symptomatic patients. Up to 83.8% of the samples tested positive for ≥1 organism, with all seven target organisms detected in at least one sample. *Ureaplasma parvum* was the most common organism detected, followed by *N*. *gonorrhoeae*, *M*. *hominis*, *U*. *urealyticum*, *T*. *vaginalis*, *C*. *trachomatis*, with *M*. *genitalium* being the least detected. All 25 samples submitted for routine antenatal Group B Streptococcal screening were positive for at least one STI organism, and one sample from sexual non-accidental injury tested positive for five different organisms.

**Conclusions:**

STIs comprise a variety of organisms in our setting, with many patients exhibiting coinfection with multiple organisms. This suggests the need for a critical evaluation of current syndromic testing and treatment guidelines so as to stem inadvertent spread of STI organisms and the development of resistance. The use of molecular testing methods may improve detection, especially in resource limited settings, providing speedy results, and thus allowing for guided therapy in only infected patients.

## Introduction

Sexually transmitted infections (STI) are some of the most commonly occurring infections globally, with countries in sub-Saharan Africa disproportionately affected [1, 2]. Populations considered higher-risk such as persons living with HIV, young women, pregnant women, men-who-have-sex-with-men (MSM), and commercial sex workers have increased incidences of STIs [2].

Infection with *Chlamydia trachomatis* is the most reported STI pathogen in the USA, followed by *Neisseria gonorrhoeae*, likely due to increased spread and testing in high-risk populations, coupled with the increased sensitivity of molecular diagnostic tests [3, 4]. Trichomoniasis is reported to be the most commonly occurring STI globally, with a significantly disproportionate burden in Sub-Saharan Africa [5–7]. An association has been reported between *T*. *vaginalis* and other STI pathogens like *N*. *gonorrhoeae*, *C*. *trachomatis*, Herpes Simplex Virus, and syphilis, linked with an increased risk of HIV infection [8, 9].

Current WHO and South African guidelines recommend syndromic management of STIs, including *N*. *gonorrhoeae* [2, 10]; however, numerous reports have emerged indicating the poor performance of this approach to accurately detect and appropriately treat these infections [11–14]. Multiplexed Nucleic Acid Amplification Tests (NAATs) are able to test for several pathogens simultaneously, reducing the number of tests and associated costs [15]. The Allplex STI Essential Assay (Seegene Inc, Korea) previously reported 100% sensitivity, and high specificity rates for seven STI organisms (*C*. *trachomatis*, *Mycoplasma genitalium*, *Mycoplasma hominis*, *N*. *gonorrhoeae*, *Trichomonas vaginalis*, *Ureaplasma parvum*, *Ureaplasma urealyticum*) [16, 17]. This assay has been internationally validated for diagnostic use, and is both Conformité Européene and in vitro diagnostics (CE-IVD) certified for use in Europe and the USA.

Currently, no broad multiplex molecular panel is used at the National Reference Laboratory, or in our setting, to detect STI organisms concurrently. The use of routine microbiological culture and microscopy, along with individual or duplex PCR is employed to detect STI organisms. The introduction of a broad multiplex assay may therefore significantly improve detection rates compared to routine testing and syndromic management, allowing for timeous and appropriate guided therapy, limiting spread and the development of resistance. This prospective observational study sought to determine the occurrence of STI, using a validated molecular assay as a diagnostic and surveillance tool in our setting. Additionally, the impact on detection rates was compared using a second in-house multiplex PCR assay for *N*. *gonorrhoeae* and *C*. *trachomatis*.

## Methods

This cross-sectional, observational, diagnostic laboratory survey of STI organisms was approved by the University of Cape Town Human Research Ethics Committee (HREC REF 408/2021), conducted according to the Declaration of Helsinki, and carried out in accordance with the required ethical guidelines and regulations. Residual diagnostic specimens and laboratory data, submitted for routine testing as clinically indicated were used, and the need for informed consent was therefore waived by the ethics committee. Patient demographic information including age, sex, sample type, and clinical diagnostic test results, ward, and clinical indications were recorded. No participant-identifying information is presented, and only laboratory and clinical staff had access to participant-identifying information as required for laboratory testing.

This study was conducted at the National Health Laboratory Services (NHLS), Groote Schuur Hospital (GSH), Cape Town, South Africa. The hospital provides tertiary and quaternary care for a large population in the City of Cape Town Metropolitan, and both the hospital and NHLS laboratory serve as a referral centre for regional and district hospitals. Urogenital swabs were submitted to the laboratory for routine STI testing or Group B streptococcus screening as clinically indicated, from 04 August 2021–03 February 2022. A total of 148 non-duplicate swabs were collected for this study, using convenience sampling over the study period.

Clinical indications for STI testing of symptomatic patients included swabs collected from patients presenting with STI syndromes (n = 56), as defined by the WHO guidelines for syndromic management [10]. Asymptomatic patients included those who did not present with typical STI-indicative symptoms, but had other indications for testing, such as pregnant women for antenatal Group B streptococcal screening (n = 25); sexual non-accidental injury (SNAI) of minors where rape was suspected (n = 5); non-specific indications which were indicative of urogenital infection but not STI-specific (n = 10); and where no clinical indication was recorded in the chart, but STI testing was requested (n = 52).

Routine diagnostic testing, according to local standard operating procedures, was performed on all swabs; this included Gram-staining, wet mount microscopy for parasites, and culture on selective and non-selective media for *N*. *gonorrhoeae*. No other tests are performed for STI pathogens in this setting. This data was extracted from the laboratory information system as needed. This data was used to evaluate their potential as diagnostic markers for infection, compared to PCR.

DNA was extracted from all 148 swabs using the Zymo Research Quick DNA Fungal/Bacterial Miniprep kit (Inqaba Biotech), according to the manufacturer’s instructions. PCR screening for STI organisms was performed, according to the manufacturer’s instructions, using the Allplex STI Essential Assay (Seegene Inc, South Korea) which is CE-IVD certified for diagnostic testing. To determine the impact of PCR assay selection on detection rates, a previously validated multiplex in-house PCR assay for *N*. *gonorrhoeae* and *C*. *trachomatis*, designed, optimised and validated for our setting using previously published primers and probes (Integrated DNA Technologies), was also evaluated [18, 19].

Fisher’s exact test, and logistic regression were used to determine associations between the participants age, clinical indications for testing, and PCR results. Positive Percent Agreement (PPA) and Negative Percent Agreement (NPA) were calculated using standard calculations, with the Allplex assay serving as the reference test. Statistical analyses were performed using STATA v16.0 and GraphPad Prism v9.0.

## Results

Of 148 swabs collected during the 6-month study period the majority of samples (91.2%) were obtained from women, from whom 25 samples submitted for Group B Streptococcal Screening had pregnancy confirmation (Table 1). The median age of men (31 y/o [Range 3–57], IQR = 20) and women (28 y/o [Range 0–75], IQR = 11) were not significantly different (p = 0.65).

**Table 1:** Participant demographics and associations.

	Number (n = 148)	Percentage	OR (PCR-positivity)	p-value	95% CI
***SEX***			**Bivariate logistic regression**		
*Male (reference category)*	13	8.78	1.63	0.486	0.41–6.42
*Female*	135	91.22			
*Confirmed pregnant*	25	18.52			
***CLINICAL INDICATION***			**Fischer’s exact test**		
*Group B Streptococcal screening*	**25**	**16.89**		**0.014**	
*Non-specific indication*	10	6.76		0.367	
*Not stated*	52	35.14		0.489	
*Sexual non-accidental injury*	5	3.38		0.185	
*STI syndrome*	56	37.84		0.250	
***PCR POSITIVITY BY INDICATION***			**Fischer’s exact test**		
*Ureaplasma parvum*	63	42.57		0.123	
*Neisseria gonorrhoeae*	56	37.84		0.298	
*Mycoplasma hominis*	51	34.46		1.0	
*Ureaplasma urealyticum*	**35**	**23.65**		**0.046**	
*Trichomonas vaginalis*	17	11.49		0.109	
*Chlamydia trachomatis*	15	10.14		0.785	
*Mycoplasma genitalium*	2	1.35		0.526	
***AGE CATEGORY (yrs.)***			**Fischer’s exact test**		
*0–10*	26	17.57	0.58	0.377	0.19–2.0
*11–16*	6	4.05	0.37	0.251	0.05–4.32
***17–30***	**54**	**36.49**	**4.89**	**0.0097**	**1.34–26.71**
*31–40*	45	30.41	1.07	1.0	0.38–3.32
*41–50*	7	4.73	0.46	0.317	0.07–5.18
*51–60*	4	2.70	0.57	0.511	0.04–31.22
*>60*	6	4.05	0.17	0.054	0.02–1.41

Greater than a third of samples submitted were from symptomatic patients presenting with STI-syndromes (n = 56). The remaining 92 samples, from asymptomatic patients, included antenatal Group B Streptococcal screening (n = 25), non-specific urogenital infections (n = 10), samples from sexual non-accidental injury of minors where rape was suspected (n = 5), and finally where no clinical indication was recorded, but STI testing was requested (n = 52).

**Fig 1:**
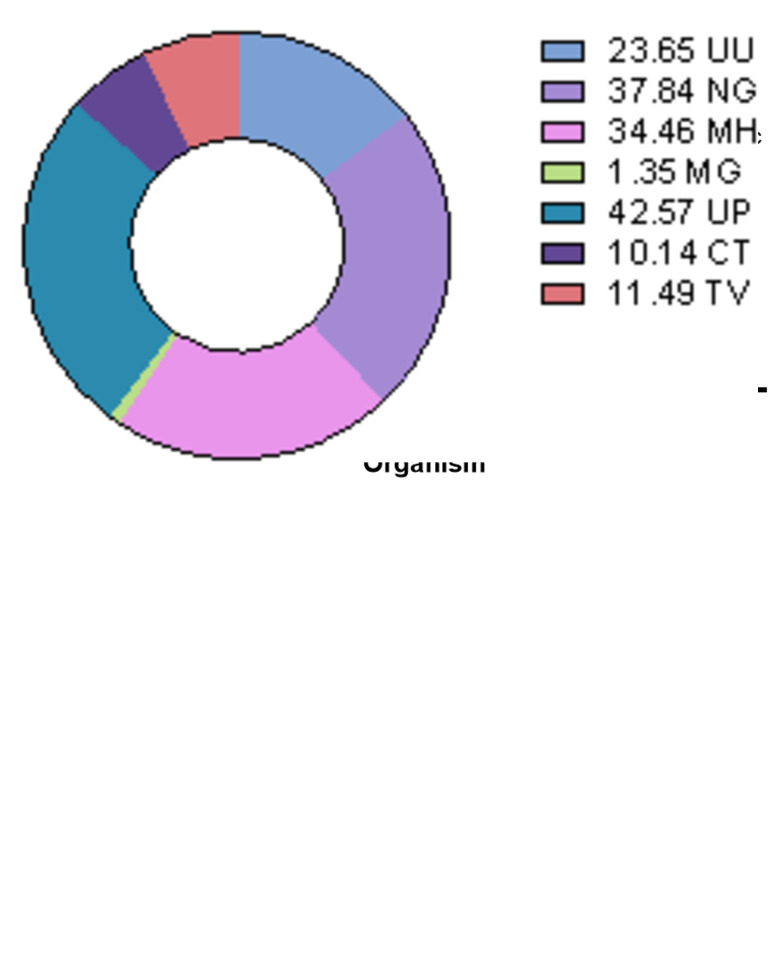
(Left) Proportion of organisms detected in symptomatic and asymptomatic patients. (Right) Pie chart of the proportion of positive samples for each target tested. UU, U. urealyticum; NG, N. gonorrhoeae; MH, M. hominis; MG, M. genitalium; UP, U. parvum; CT, C. trachomatis; TV, T. vaginalis.

Using the Allplex STI essential assay, all seven targets included in the panel were detected in at least one of the samples tested, with 83.8% (124/148) of samples testing positive for one or more organism. *Ureaplasma parvum* was the most commonly detected organism (42.6%), followed by *N*. *gonorrhoeae*, *M*. *hominis*, *U*. *urealyticum*, *T*. *vaginalis*, *C*. *trachomatis*, with *M*. *genitalium* the least common (Table 1 and Fig 1 right, S1 Table in S1 File).

Of all samples tested, 36.5% (54/148) were positive for a single organism, where *N*. *gonorrhoeae* was the most commonly occurring pathogen in mono-infection (19.0%), followed by *U*. *parvum* (10.1%), *M*. *hominis* (4.1%), *U*. *urealyticum* (2.7%), and *C*. *trachomatis* (0.7%). 16.2% of samples tested negative for all assayed organisms (S1 Fig in S1 File).

There were several combinations of co-infections with multiple organisms (S1 Fig in S1 File). 27.0% of samples were positive for two organisms, while 11.5% tested positive for three organisms, 7.4% for four, and 1.4% with five organisms. *M*. *hominis* and *U*. *parvum* were the most commonly occurring organisms in co-infections (36.3% each), followed by *U*. *urealyticum* (25.0%) and *N*. *gonorrhoeae* (22.6%), with each of the seven organisms in the assay detected in combination with at least one other organism (S1 Fig in S1 File). *M*. *hominis* and *T*. *vaginalis* only occurred in co-infection.

Although no significant difference was observed overall for PCR-positivity between symptomatic and asymptomatic participants, asymptomatic participants were significantly more likely to test positive for *U*. *urealyticum* (p = 0.046) (Fig 1 left).

Of the samples from symptomatic patients, only 44/56 (78.6%) were PCR-positive for one or more of the organisms assayed, compared to 80/92 (84.0%) of asymptomatic patients. Symptomatic patients were predominantly positive for *N*. *gonorrhoeae*, *M*. *hominis*, and *U*. *parvum*, whereas asymptomatic cases also predominantly included *U*. *urealyticum* (Fig 1 left).

Of the sexual non-accidental injury samples submitted, 3/5 (60.0%) were positive with 1, 2, and 5 organisms, respectively. All samples submitted for Group B streptococcal screening (n = 25) tested positive for at least one organism (p = 0.014). *M*. *genitalium* was only detected in two samples, both submitted for Group B streptococcal screening, and was in co-infection with both *M*. *hominis*, and either *U*. *urealyticum*, or *U*. *parvum* (S1 Fig in S1 File).

**Fig 2:**
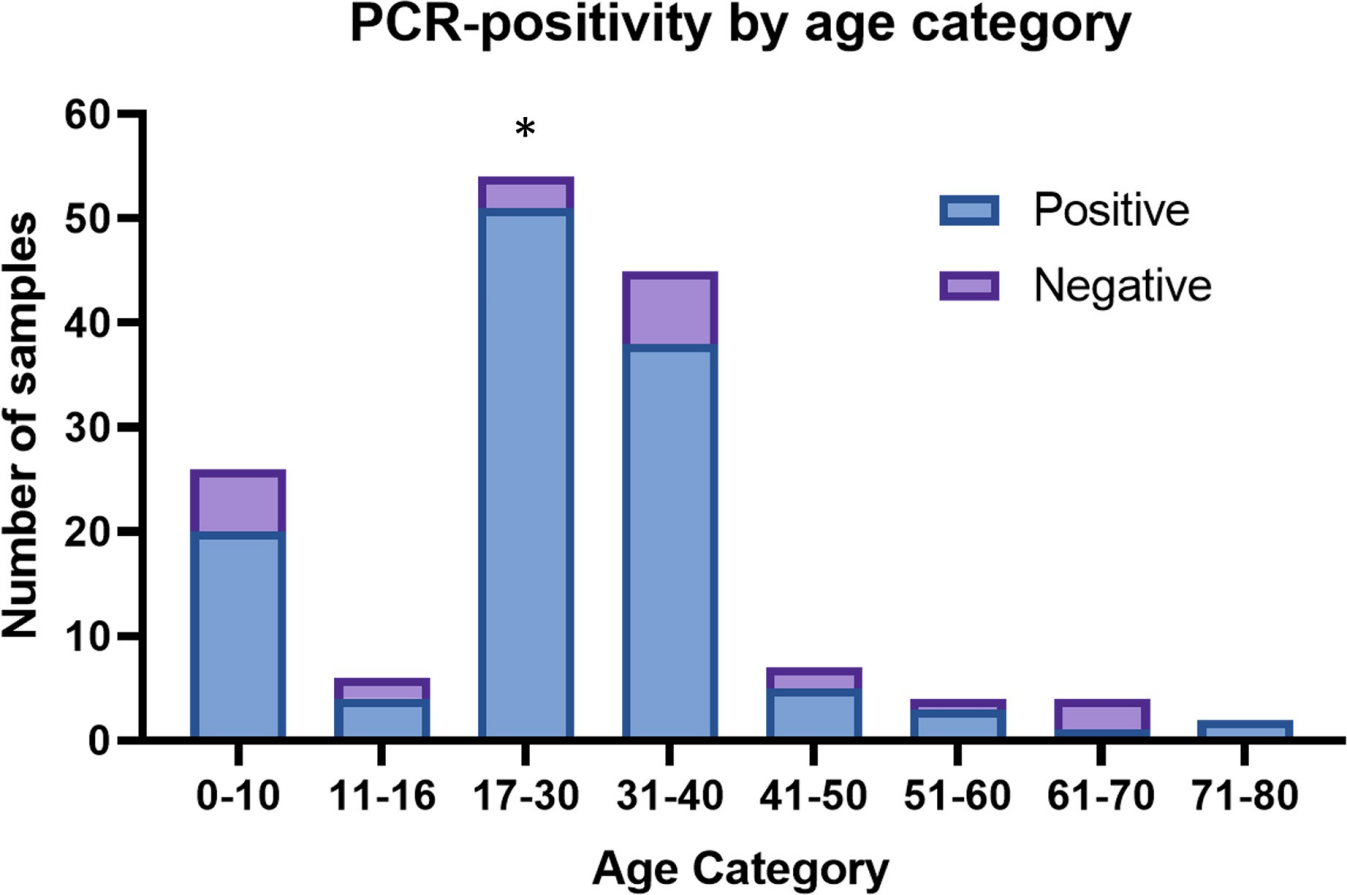
Distribution of PCR-positive results by age category.

Most samples received were in the 0-10-, 17-30- and 31-40-year-old age categories (Fig 2). Participants who were 17–30 (OR 4.89, 95% CI 1.34–26.71; p = 0.0097) years old were significantly more likely to test PCR-positive for any of the organisms assayed (Fig 2 and Table 1). No significant differences were noted for the other categories, and this may be attributed to the small number of samples submitted in these age categories.

Interestingly, in the 0–10 y/o age category, 20/26 (77%) samples tested positive for one or more organisms. Excluding the SNAI cases, which were more likely to have had exposure to these organisms in this age category, the remaining 19/21 (90%) samples tested positive for *N*. *gonorrhoeae* (n = 13), or one of the less common STI associated organisms, including *U*. *parvum* (n = 4), *M*. *hominis* (n = 4), *U*. *urealyticum* and *T*. *vaginalis* (n = 1). These mostly occurred as co-infections with each other.

Compared to the PCR results, routine laboratory testing results were investigated as potential markers of STI. *N*. *gonorrhoeae* mono or co-infection was detected in 56 (37.8%) of samples using the Allplex assay. Only 3/56 (5.4%) of these samples were culture-positive for *N*. *gonorrhoeae*, and 1/3 of these samples was from a symptomatic patient. The remaining 2/3 culture-positive samples were from asymptomatic patients where no clinical indication was recorded, and only 1/2 of these also had Gram negative diplococci on microscopy. An additional sample was positive for Gram negative diplococci on microscopy, but this sample was culture negative for *N*. *gonorrhoeae*, and was PCR-positive for *U*. *parvum* alone (S2 Fig in S1 File). Of the 17 *T*. *vaginalis* PCR-positive samples, only one was positive on wet mount microscopy.

The results of the Allplex assay for detecting *N*. *gonorrhoeae* and *C*. *trachomatis* were compared to a laboratory validated, in-house multiplex PCR assay, targeting these organisms [18]. Several discrepancies were noted between the two assays, with poor positive percent agreement for both organisms. Negative percent agreement was better, with 100% correlation for *C*. *trachomatis*. The overall percent agreement was also poor for *N*. *gonorrhoeae*, but markedly better for *C*. *trachomatis* (Table 2).

**Table 2:** Assay comparative values.

	PPA (n)	NPA (n)	OPA (n)
*N*. *gonorrhoeae*	58.97% (46/78)	85.71% (60/70)	71.62% (106/148)
*C*. *trachomatis*	78.95% (15/19)	100% (129/129)	97.30% (144/148)

PPA, Positive Percent Agreement; NPA, Negative Percent Agreement; OPA, Overall Percent Agreement.

## Discussion

This study, which aimed to describe the occurrence of STI organisms at an academic and referral diagnostic laboratory and hospital, documented a positive result for at least one of the seven STI-organisms included in the Allplex STI PCR assay, in most of the collected samples. This was alarming considering there were no study specific *a priori* selection criteria for high-risk populations, and all swabs from both symptomatic and asymptomatic patients were included. This was further reflected in the higher proportion of positive samples in the asymptomatic patients. However, since the swabs were submitted for routine urogenital investigations following clinical investigation of urogenital problems, this may have presented a high pre-test probability for an STI. Considering the NHLS laboratory serves as a referral centre for regional and district hospitals within a referral area of 755 km^2^, with an estimated low to lower-middle-income population of 1.85 million, the results are reflective of a large population in the region [20].

With the introduction of the WHO and CDC guidelines for the management of STIs [10, 21], the available data for laboratory confirmed infections and antimicrobial susceptibility testing are sparse, making comparisons of estimated prevalence rates complex, relying primarily on national surveillance estimates. Most studies focused on high-risk populations or young women, since they are disproportionately at a higher risk for STI [2, 4]. Most swabs submitted for testing in this study were received from women, and the median age correlated well with international studies where STI-prevalence was highest [13, 22]. National and regional surveillance studies describing STI prevalence rates are limited and vary significantly based on demographic, socio-economic, and geographic factors, as well as national policies for prevention and treatment. A 2018 study, modelling STI prevalence rates in South Africa over 30-years, indicated an adjusted *N*. *gonorrhoeae* prevalence estimate of 6.6% for women and 3.5% for men, and *C*. *trachomatis* prevalence of 14.7% and 6.0% for women and men respectively, which were among the highest reported globally [13].

This study indicated a high burden of infection in both men and women, in our setting. Interestingly, infection rates in asymptomatic patients were higher than those presenting with symptoms indicative of an STI syndrome. This may be due the fact that most STIs are asymptomatic, and with the use syndromic management guidelines asymptomatic infections may inadvertently contribute to the spread of STI. Previous studies have indicated that the risk for STI in high HIV-burdened regions are increased and is associated with lower education levels, several sex partners, drug and alcohol use, and early sexual debut [23].

Samples submitted for Group B streptococcal screening are collected from pregnant women in the final stages of pregnancy to determine whether antibiotic prophylaxis should be administered during delivery to prevent vertical Group B streptococcal infection in the new-born infant, which may cause severe morbidity and even mortality, according to the CDC guidelines [24, 25]. Interestingly, only two Group B streptococcal screening samples were culture positive for Group B streptococci, whereas they all tested positive for one or more STI organisms. Of these, 40.0% (10/25) were *N*. *gonorrhoeae* positive, 4/25 (16.0%) *T*. *vaginalis* positive, and 3/25 (12.0%) were *C*. *trachomatis* positive. Further, *M*. *genitalium* was only detected in Group B streptococcal screening samples, and was in co-occurrence with both *M*. *hominis* and either *U*. *urealyticum* or *U*. *parvum*. These findings highlighted a potential high-risk population group, who would otherwise have been undiagnosed, and which should be considered for comprehensive antenatal STI screening in our setting. This is especially important as studies have indicated an association between STI infections and pre-term birth, transmission to the foetus or new-born baby, or even stillbirth [26].

There are few studies investigating the occurrence of the less common STI associated organisms, and some contention exists on their occurrence and the association with true infection, and subsequently whether or not to treat. There is no clear evidence that these organisms cause infection independently of confirmed STI pathogens, or whether they are co-factors for infection, or simply vaginal commensals, though their presence has been linked to complications during pregnancy [27, 28]. Interestingly, this study indicated a high level of the less common STI associated organisms in the 0–10 y/o age category. This is rather interesting, since in the absence of rape these organisms are not expected to occur in the genital tract unless they are commensals. Future work could focus on studying the associations between these organisms and infection or disease in a cohort which is naïve to sexual intercourse.

The detection of *U*. *parvum*, *U*. *urealyticum*, *M*. *hominis*, and *M*. *genitalium* in this study were all markedly higher than those determined in an Italian study on women with vaginitis or pregnancy complications, which used the same PCR assay [29]. This is further highlighted in this study where although *M*. *hominis*, *U*. *urealyticum*, and *U*. *parvum* were identified in mono-infection in this study, albeit at lower rates, these three organisms were the most commonly occurring organisms in co-infections, with *M*. *hominis* and *T*. *vaginalis* only detected in coinfection with other pathogens [8]. Since no HIV data was collected in this study, it is not possible to correlate this to an increased risk of HIV acquisition or virus shedding, which has been noted in previous studies [9].

Interestingly, about one fifth of the samples submitted for suspected STI-syndromes were negative for all organisms tested. This reflects a large proportion of patients where needless antibiotic therapy may have been initiated based on the current syndromic testing guidelines [10].

Most alarmingly, more than half of the samples submitted from sexual non-accidental injury were positive for one of the targets, with one sample testing positive for five different organisms, likely a result of high-risk unprotected sexual abuse. The use of a multiplex molecular assay would provide swift results for early therapeutic interventions, increase detection sensitivity for multiple organisms, and provide reliable results for further legal proceedings.

The utility of routine STI laboratory testing results as markers of STI in resource limited setting are of great value, in the absence of more sensitive methods. Compared to PCR, the results obtained from the routine microbiological investigations; selective culture, Gram stain, and wet mount microscopy performed extremely poor as markers of infection, with only three samples testing positive for *N*. *gonorroheae*, and one for *T*. *vaginalis*. This is not surprising considering the fastidious nature of *N*. *gonorroheae*, that infections with low bacterial load may be below the detection limit for these methods, and that no other STI testing is performed in this setting. Routine microbiological testing results should therefore be used judiciously for STIs, and should be correlated with other clinical data, and confirmed with molecular testing when not feasible for routine screening.

Considering the debatable role of some of the STI organisms in infection, they may be considered for secondary testing in persistent infections, following negative screening for primary STI pathogens, as is recommended in several countries [30]. The added value of a multiplex molecular assay for selected STI organisms, especially in a resource limited setting, would significantly improve the current detection rates and result time [31, 32].

Since both assays used in this study were fully validated for diagnostic testing, the same DNA eluates and template volumes were used to compare the performance of the two assays in a head-to-head comparison. The in-house PCR assay produced several discordant results compared to the reference Allplex assay. Even though the in-house assay produced higher positivity rates, test reproducibility was poor, reflected by the low PPA, NPA, and higher CT values. The in-house assay has been shown to detect very low levels of carryover of amplicons in our setting, which is not reproducible on repeat testing. The Allplex assay utilises an uracil-N-glycosylase–deoxy-uridine-triphosphatase (UNG-dUTP) system to eliminate the detection of amplicon carryover contamination. The specificity of the in-house assay was confirmed by testing *N*. *meningitidis* and *N*. *mucosa*, which were both undetected. Cross-reactivity is therefore less likely to have produced the higher positivity rate with this assay, but this cannot be ruled out as other related species, which were not evaluated, may be detected using this assay. The Allplex assay was validated using one hundred and twenty-three different STI organisms, including eight different *Neisseria* spp. and no cross reactivity was noted for any of the organisms tested.

Comparative data of the two assays indicated poor correlation, with good negative percent and overall agreement for *C*. *trachomatis*, although this may be due to the low number of positive results for this organism. Care should therefore be taken when selecting diagnostic assays for STI testing, which are known to cross react with other species and may be impacted by interfering substances, and cross contaminating nucleic acids. The use of a certified assay, with reliable assay performance should be favoured [33].

Limitations of this study include the small and heterogenous sample size, as the associations described may not hold true at a population level, but may be worth investing in a population surveillance study, especially with respect to the higher risk groups identified in this study. There is a paucity in the current regional and National prevalence data, making comparisons of these findings challenging.

## Conclusions

This study highlights the burden of STI organisms, documenting notable coinfection with multiple organisms, among our patient cohort. The rates of detection in asymptomatic patients, specifically pregnant women, and sexual assault, indicate who may benefit from routine testing using a reliable PCR assay. Syndromic testing and treatment guidelines may not sufficiently target vulnerable populations, and the use of molecular testing methods may improve detection, especially in resource limited settings, providing faster results, and thus allowing for guided therapy in infected patients.

## Supporting information

S1 File (DOCX)

S1 Dataset (XLSX)

## List of abbreviations

STI: Sexually transmitted infection
HIV: Human Immunodeficiency Virus
MSM: Men-who-have-Sex-with-Men
WHO: World Health Organisation
USA: United States of America
NAAT: Nucleic Acid Amplification Tests
NHLS: National Health Laboratory Service
NYC: New York City
BBA: Boiled Blood Agar
GBS: Group B streptococcal
THB: Todd-Hewitt Broth
DNA: Deoxyribonucleic acid
PCR: Polymerase Chain Reaction
CE: Conformité Européenne
IVD: In vitro Diagnostic
yrs: Years
OR: Odds Ratio
CI: Confidence Interval
n: Number
UU: *Ureaplasma urealyticum*
NG: *Neisseria gonorrhoeae*
MH: *Mycoplasma hominis*
MG: *Mycoplasma genitalium*
UP: *Ureaplasma parvum*
CT: *Chlamydia trachomatis*
TV: *Trichomonas vaginalis*
SNAI: Sexual non-accidental injury
UNG-dUTP: Uracil-N-glycosylase–deoxy-uridine-triphosphatase
