# Supplementary material for: Evaluating the utility of the Allplex STI Essential Assay to determine the occurrence of urogenital sexually transmitted infections among symptomatic and asymptomatic patients in Cape Town, South Africa
Source: PLoS One. 2023 Nov 29;18(11):e0292534. doi: 10.1371/journal.pone.0292534 (PMC10686431; doi:10.1371/journal.pone.0292534)
Supplement: S1 File — (DOCX) [file pone.0292534.s001.docx]

**Supplementary data**

***S Fig 1. Proportions of mono and co-infections.*** *U. urealyticum; NG, N. gonorrhoeae; MH, M. hominis; MG, M. genitalium; UP, U. parvum; CT, C. trachomatis; TV, T. vaginalis.*

***S Fig 2. Venn diagram of PCR-positive samples, culture, and gram stain.***

S Table 1. Number of organisms detected in symptomatic and asymptomatic patients.

| **Organism** | **Symptomatic** | **Asymptomatic** |
| --- | --- | --- |
| Ureaplasma urealyticum | 8 | 27 |
| Neisseria gonorrhoeae | 18 | 38 |
| Mycoplasma hominis | 19 | 32 |
| Mycoplasma genitalium | 0 | 2 |
| Ureaplasma parvum | 19 | 44 |
| Chlamydia trachomatis | 5 | 10 |
| Trichomonas vaginalis | 3 | 14 |
